# Effects of dasatinib on EphA2 receptor tyrosine kinase activity and downstream signalling in pancreatic cancer

**DOI:** 10.1038/sj.bjc.6604676

**Published:** 2008-09-16

**Authors:** Q Chang, C Jorgensen, T Pawson, D W Hedley

**Affiliations:** 1 Division of Applied Molecular Oncology, Ontario Cancer Institute/Princess Margaret Hospital, Toronto, Ontario, Canada M5G 2M9; 2 Samuel Lunenfeld Research Institute, Mount Sinai Hospital, Toronto, Ontario, Canada M5G 1X5; 3 Department of Molecular and Medical Genetics, University of Toronto, Toronto, Ontario, Canada M5S 1A8; 4 Department of Medical Oncology and Hematology, Ontario Cancer Institute/Princess Margaret Hospital, Toronto, Ontario, Canada M5G 2M9; 5 Department of Medical Biophysics, University of Toronto, Toronto, Ontario, Canada M5S 1A8

**Keywords:** EphA2, receptor tyrosine kinase, pancreatic cancer, dasatinib, xenografts

## Abstract

Eph receptors constitute the largest family of receptor tyrosine kinases in the human genome. EphA2 is one prominent member that is overexpressed and functionally altered in many invasive cancers, including pancreatic cancer. Dasatinib, which is a multi-targeted kinase inhibitor mainly developed for Bcr-Abl and Src family kinases, has recently been shown to have significant activity against EphA2. As selective small molecule EphA2 inhibitors are not currently available, we investigated the therapeutic potential to target EphA2 by dasatinib in pancreatic cancer cell lines. Using *in vitro* kinase assays, we found that EphA2 receptor tyrosine kinase was inhibited directly by dasatinib in a dose-dependent manner. Stimulation with ephrinA1 produced rapid increases of EphA2 phosphorylation that were inhibited by dasatinib, although the effects on activation of downstream signalling differed among the pancreatic cancer cell lines. Dasatinib also inhibited ligand-induced binding of EphA2 to the ubiquitin ligase Cbl, and the internalisation and degradation of EphA2, suggesting that these processes are dependent on kinase activity. Treatment with dasatinib decreased EphA2 phosphorylation in BxPC-3 xenografts, suggesting that dasatinib might have activity in pancreatic cancer due to EphA2 inhibition, besides its effects on Src.

The Eph family of receptor tyrosine kinases (RTKs), with at least 14 distinct members in mammals, constitutes the largest subfamily of RTKs (Pasquale, 2004). Eph RTKs are divided into two subclasses (EphA and EphB) based on sequence similarity and their preferential binding to ligands, which are tethered to the cell surface either by a glycosylphosphatidylinositol-anchor (ephrinA) or by a single transmembrane domain (Kullander and Klein, 2002). Upon ligand binding, Eph receptors multimerise and become phosphorylated (Dodelet and Pasquale, 2000). Eph RTKs are increasingly understood to have significant functions in disease and development (Zantek *et al*, 1999). In normal development, Eph receptors frequently control the compartmentalisation of cells in complex tissues such as the vascular system, brain and intestinal epithelium, through their ability to mediate cellular repulsion and adhesion. In tumourigenesis, they have been implicated in cellular transformation, metastasis and angiogenesis (Nakamoto and Bergemann, 2002). For example, B type receptors appear to suppress the invasiveness of colon cancer cells (Batlle *et al*, 2005), whereas A type receptors such as EphA2 may have a pro-oncogenic effect.

EphA2 critically controls many aspects of cell behaviour (Kinch and Carles-Kinch, 2003). Epithelial cells normally form stable linkages with adjacent cells, and express low levels of EphA2 that are enriched within intercellular junctions (Zantek *et al*, 1999). This localisation favours stable ligand binding, and indeed, EphA2 on normal epithelial cells is autophosphorylated (Zantek *et al*, 1999; Miao *et al*, 2001). In contrast, malignant cells generally show unstable cell–cell contacts (Kinch and Burridge, 1995), and a consequence of this change is that the high levels of EphA2 on malignant cells fail to bind ligand and thus become diffusely distributed over the cell surface (Zantek *et al*, 1999; Macrae *et al*, 2005). Thus, EphA2 function can be altered in malignant cells (Kinch and Carles-Kinch, 2003). EphA2 is also frequently overexpressed and functionally altered in many invasive cancers (Walker-Daniels *et al*, 2003). For example, high levels of EphA2 have been documented in metastatic melanoma, as well as cancers of the mammary gland, cervix, ovary, prostate, colon, lung, kidney, esophagus and pancreas (Easty *et al*, 1995; Ogawa *et al*, 2000; Zantek *et al*, 2001; Zelinski *et al*, 2001; Nakamoto and Bergemann, 2002; Kinch *et al*, 2003; Miyazaki *et al*, 2003; Ireton and Chen, 2005; Mudali *et al*, 2006).

However, despite the strong correlation of EphA2 receptor expression with malignant phenotypes, the mechanisms by which EphA2 contributes to tumour cell malignancy are far from clear (Fang *et al*, 2005). Some evidence supports the idea that EphA2 receptor phosphorylation is not necessary to confer kinase activity and tumorigenicity (Zantek *et al*, 1999; Walker-Daniels *et al*, 2002), or is even tumour suppressive (Guo *et al*, 2006). Other data suggest that EphA2 receptor phosphorylation may be important in conferring the oncogenic potential (Ogawa *et al*, 2000; Brantley *et al*, 2002; Dobrzanski *et al*, 2004). Hence, EphA2 represents a therapeutic target for novel anticancer agents.

Dasatinib is an oral dual Bcr/Abl and Src family kinases inhibitor, approved for use in patients with chronic myelogenous leukaemia and currently tested as an Src inhibitor (Talpaz *et al*, 2006). In a recent report, Huang *et al* (Huang *et al*, 2007) showed that EphA2 was highly expressed in dasatinib-sensitive cell lines and that EphA2 was also inhibited by dasatinib. Furthermore, EphA2 and several other members of Eph RTKs have been shown as targets of dasatinib by gene expression and a chemical proteomic profiling approach (Bantscheff *et al*, 2007; Rix *et al*, 2007; Wang *et al*, 2007). As EphA2 is frequently overexpressed in pancreatic cancer (Duxbury *et al*, 2004; Mudali *et al*, 2006), and selective small molecule EphA2 inhibitors are not currently available, we investigated the therapeutic potential to target EphA2 by dasatinib in several pancreatic cancer cell lines as well as in BxPC-3 xenografts.

## Materials and methods

### Cell lines

The human pancreatic cancer cell lines BxPC-3, PANC-1, MIA PaCa-2 and human embryonic kidney (HEK) 293 cell line were obtained from American Type Culture Collection (Manassas, VA, USA). BxPC-3 was grown in RPMI-1640 with 10% fetal bovine serum (FBS). PANC-1 was grown in Dulbecco's modified Eagle's medium (DMEM)-H21 with 10% FBS. MIA PaCa-2 was grown in DMEM-H21 with 10% FBS and 2.5% horse serum. HEK-293 was grown in DMEM with 10% FBS. Cells were maintained at 37°C under standard cell culture conditions.

### Reagents and antibodies

Mouse monoclonal antibodies against phosphotyrosine (P-Tyr-100), phospho-STAT3 (Tyr705) as well as rabbit polyclonal antibodies against phospho-Src (Tyr416), phospho-FAK (Tyr576/577), phospho-FAK (Tyr925), phospho-Akt (Ser473), phospho-Paxillin (Tyr118), phospho-p44/42 MAPK (Thr202/Tyr204) and antibodies directed against their nonphosphorylated counterparts, were purchased from Cell Signaling Technology (Beverly, MA, USA). Mouse monoclonal antibody against phospho-STAT3 (Ser727) was from BD Biosciences (Mississauga, Ontario, Canada). Rabbit polyclonal antibodies directed against EphA2 and Cbl were obtained from Santa Cruz Biotechnology Inc. (Santa Cruz, CA, USA). A mouse monoclonal antibody against EphA2 and recombinant mouse ephrin-A1/Fc chimera were from R&D Systems Inc. (Minneapolis, MN, USA). The secondary antibodies for western blots (anti-mouse and anti-rabbit IgG antibodies) were from Amersham Biosciences (Buckinghamshire, United Kingdom). Protein G-Sepharose was from Sigma-Aldrich Canada Ltd. (Oakville, Ontario, Canada). AffiniPure goat anti-human IgG, Fc_*γ*_ fragment-specific was from Jackson ImmunoResearch laboratories Inc. (West Grove, PA, USA). [*γ*-^32^P]ATP was from New England Nuclear (Boston, MA, USA).

### Drug preparation

Dasatinib was purchased from Toronto Research Chemicals (North York, Ontario, Canada). PP2 was purchased from Calbiochem (San Diego, CA, USA). Dasatinib and PP2 used for tissue culture were both dissolved in DMSO, aliquoted, and stored at appropriate temperature. Dasatinib was prepared freshly as a 12.5 mg ml^−1^ suspension in 80 mM sodium citrate/citric acid buffer, pH 3.0 for oral gavage *in vivo*.

### *In vitro* kinase assays

*In vitro* autophosphorylation assays were essentially performed as described earlier (Holland *et al*, 1997). HEK-293 cells were transfected with cDNA-encoding human EphA2 or human EphB2. Cells were lyzed 20 h later and EphA2 or EphB2 was immunoprecipitated. Immunoprecipitated EphA2 or EphB2 was washed in lysis buffer [50 mmol l^−1^ HEPES (pH 7.5), 10% glycerol, 1% Triton X-100, 150 mmol l^−1^ NaCl, 1 mmol l^−1^ EGTA, 1.5 mmol l^−1^ MgCl_2_, 100 mmol l^−1^ NaF, 10 mmol l^−1^ Na_4_P_2_O_7_·10H_2_O, 1 mmol l^−1^ Na_3_VO_4_] and kinase reaction buffer, incubated with dasatinib (0, 20, 100 and 200 nM, final DMSO concentration 0.05% in all reactions) at room temperature for 10 min and then incubated with 10 *μ*Ci of [*γ*-^32^P]ATP at room temperature for 30 min. The reactions were terminated by the addition of 6 × Laemmli sample buffer and boiling. After resolving samples by 10% SDS–PAGE, the gel was stained with Coomassie blue to check for equal loading, then dried and exposed.

### Cell proliferation assay

BxPC-3, PANC-1 and MIA PaCa-2 cells were cultured in 96-well plates at 5000 cells per well in 100 *μ*l complete medium and then pretreated with 0, 25, 50, 100 and 200 nM dasatinib for 48 h. According to the manufacturer's instructions, viable cell number was determined using a colorimetric method based on the cellular reduction of the tetrazolium compound 3-(4,5-dimethylthiazol-2-yl)-5-(3-carboxymethoxyphenyl)-2-(4-sulfophenyl)-2H-tetrazolium (MTS) into a soluble coloured formazan product (CellTiter 96 Aqueous One Solution Cell Proliferation Assay, Promega Corp., Madison, WI, USA).

### Cell cycle analysis

Subconfluent BxPC-3, PANC-1 and MIA PaCa-2 cells were treated with 0, 25, 50, 100 and 200 nM dasatinib for 24 h, and then permeabilised with Triton X-100, treated with ribonuclease, and stained with 50 *μ*g ml^−1^ propidium iodide. DNA histograms were analysed using ModFit LT™ (Verity, Topsham, ME, USA).

### Ligand stimulation, immunoblotting and immunoprecipitation

Cells in logarithmic growth phase were serum-starved (0.5% serum) overnight, stimulated with preclustered 2 *μ*g ml^−1^ ephrinA1-Fc for the indicated times and then processed for immunoblotting and immunoprecipitation. Briefly, cells were washed with ice-cold PBS and lysed in lysis buffer containing protease inhibitor cocktail tablets (Roche Diagnostics, Laval, Quebec, Canada) for 1 h on ice. Equivalent amounts of protein (assayed with bicinchoninic acid protein assay from Pierce Biotechnology Inc., Rockford, IL, USA) were separated on 10% SDS–PAGE gels. Proteins were transferred to polyvinylidene difluoride membranes (Millipore, Bedford, MA, USA) and probed with the appropriate antibodies according to the manufacturer's instructions. For immunoprecipitation, EphA2 or Cbl was immunoprecipitated from 200–500 *μ*g cleared lysates using anti-EphA2 or Cbl antibody plus protein G-Sepharose. Products were fractionated as above and blots probed with anti-phosphotyrosine or anti-EphA2 antibody. Blots were stripped and re-probed with anti-EphA2 or anti-Cbl antibody. Detection was conducted using SuperSignal West Pico chemiluminescent substrate kits (Pierce Biotechnology Inc., Rockford, IL, USA) or enhanced chemoluminescence Plus detection reagents (GE Healthcare, Piscataway, NJ, USA).

### Immunofluorescence microscopy

Cells were grown on glass chamber slides coated with collagen (125 *μ*g ml^−1^, BD Biosciences, Mississauga, Ontario, Canada). For staining, cells were fixed with PBS containing 4% paraformaldehyde for 15 min at room temperature, permeabilised with 0.2% Triton X-100, then blocked with PBS containing 10% FBS. After blocking, cells were incubated with diluted monoclonal antibody overnight at 4°C followed by Cy3-conjugated goat anti-mouse IgG (Jackson ImmunoResearch, West Grove, PA, USA) at room temperature in the dark for 1 h. Control experiments with secondary antibody alone were performed to confirm the absence of background staining. Between each step, cells were washed with PBS. After final washes, slides were coverslipped with Vectashield Mounting Medium with DAPI and examined by confocal microscopy.

### BxPC-3 xenografts

Animal experiments were done in the animal facility of the Princess Margaret Hospital, operated under the guidelines approved by the Canadian Council for Animal Care. BxPC-3 cell suspension (∼0.5 ml; containing approximately 2 × 10^6^ cells) was injected subcutaneously into the left flank of 6-week-old male severe combined immunodeficient mice. After 4 weeks, the tumours became obviously visible. Five groups of three tumour-bearing mice were treated with a single dose of dasatinib (50 mg kg^−1^) or vehicle control (80 mM sodium citrate/citric acid buffer, pH 3.0) by oral gavage, and killed at 2, 4, 8, and 24 h. Each tumour was rapidly dissected from the surrounding tissues and cut into pieces that were snap frozen in liquid nitrogen, fixed in formalin for 24 h then paraffin-embedded, or homogenised in 1 ml lysis buffer for western blot and immunoprecipitation.

## Results

### EphA2 receptor tyrosine kinase activity is inhibited directly by dasatinib

**Figure 1:**
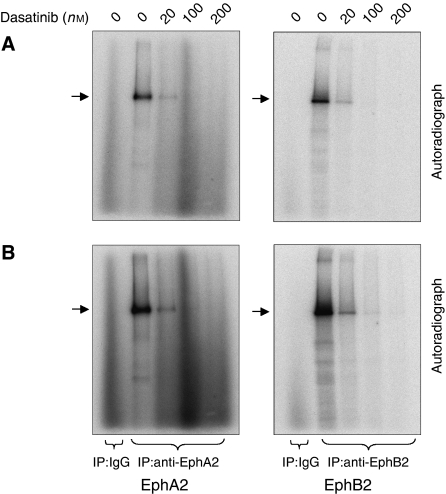
EphA2 receptor tyrosine kinase activity is inhibited directly by dasatinib. HEK-293 cell lysates transfected with EphA2 or EphB2 constructs were immunoprecipitated with anti-EphA2 or anti-EphB2 antibody and kinase assays were performed in the presence of increasing amounts of dasatinib. Inclusion of dasatinib resulted in decreased autophosphorylation. The same gel was exposed for 5 h (**A**) and then exposed for 20 h (**B**).

To determine whether the EphA2 receptor tyrosine kinase activity is inhibited by dasatinib directly or whether the ability of dasatinib to suppress EphA2 activity is an indirect effect of Src inhibition, we performed *in vitro* autophosphorylation assays. HEK-293 cells expressing EphA2 were immunoprecipitated with anti-EphA2 antibody and kinase assays were performed in the presence of increasing amounts of dasatinib. Reduced autophosphorylation was observed in a dose-dependent manner following addition of dasatinib. Interestingly, dasatinib was also found to inhibit EphB2 directly at similar concentrations (Figure 1).

### *In vitro* anti-tumour activity of dasatinib

**Figure 2:**
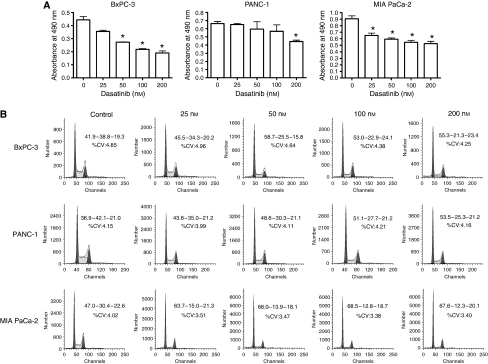
*In vitro* anti-tumour activity of dasatinib. BxPC-3, PANC-1 and MIA PaCa-2 cells were pretreated with 0, 25, 50, 100 and 200 nM dasatinib for 24 or 48 h. (**A**) Effects of 48 h treatment with dasatinib on the *in vitro* growth of BxPC-3, PANC-1 and MIA PaCa-2 cells at the indicated concentrations were measured by MTS assay. ^*^Statistically significant from control (*P*<0.05). (**B**) Single parameter DNA histograms showed the cell cycle effects of 24 h treatment with dasatinib at the indicated concentrations. Percent cells in G1-S-G2/M phase are given. CV, coefficient of variation.

As shown in Figure 2A, there was a dose-dependent decrease in cell numbers following 48 h treatment with dasatinib in all three cell lines, with MIA PaCa-2 and BxPC-3 showing greater sensitivity than PANC-1. This was associated with a corresponding decrease in the percentages of cells in S phase, as shown in Figure 2B.

### EphA2 activation affects downstream signalling

**Figure 3:**
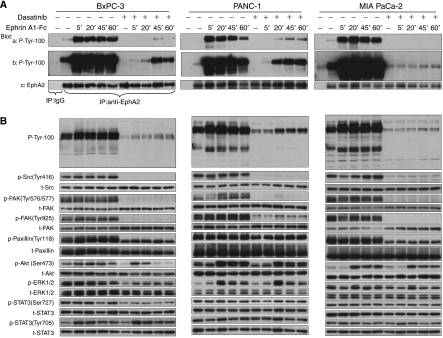
Inhibition of EphA2 receptor tyrosine kinase signalling in pancreatic cancer cell lines. Around 90% confluent serum-starved BxPC-3, PANC-1 and MIA PaCa-2 cells were pretreated with or without 200 nM dasatinib for 2 h prior to 2 *μ*g ml^−1^ ephrinA1-Fc stimulation for the indicated periods of time. Cells were harvested at various time points. (**A**) Cell lysates were immunoprecipitated with anti-EphA2 antibody, analysed by phosphotyrosine (P-Tyr-100) and EphA2 immunoblots. The same membrane was exposed for 45 s (a) and then 10 min (b) using ECL Plus. EphA2 reprobing (c) served as the loading control. (**B**) Cell lysates were analysed by western blot using antibodies directed against phosphotyrosine (P-Tyr-100), p-Src (Tyr 416), Src, p-FAK (Tyr 576/577, Tyr 925), FAK, p-Paxillin (Tyr 118), Paxillin, p-Akt (Ser 473), Akt, p-ERK1/2 (Thr 202/Tyr 204), ERK1/2, p-STAT3 (Ser 727, Tyr 705), STAT3.

In all three pancreatic cancer cell lines, low basal levels of EphA2 tyrosine phosphorylation were detected in the absence of ligand, and these showed large increases following ligand stimulation (Figure 3A). As revealed by immunoblotting of total cell lysates with a phosphotyrosine antibody (Figure 3B), the tyrosine phosphorylation of several cellular proteins was significantly induced following ephrinA1-Fc stimulation. We also probed these lysates with phospho-specific antibodies to a number of cellular signalling proteins. BxPC-3 cells showed increased Src phosphorylation at Tyr 416 compared with PANC-1 and MIA PaCa-2 cells and we did not observe obvious responses upon ligand binding in these cells. In contrast, Src and focal adhesion kinase (FAK) showed transient dephosphorylation in PANC-1 and MIA PaCa-2 cells, consistent with previous reports (Miao *et al*, 2000). However, paxillin, a downstream substrate of FAK, showed no obvious responses to EphA2 activation in any of the three cell lines. Interestingly, our data also showed that Akt phosphorylation at Ser 473 was increased following ligand stimulation. EphA2 activation inhibited p44/42 mitogen-activated protein kinase (MAPK) phosphorylation at Thr 202/Tyr 204 (phosphorylated extracellular signal-regulated kinase, p-ERK1/2) in BxPC-3, yet p-ERK1/2 level was increased at the 20 min ephrinA1-Fc-binding time point in PANC-1 and MIA PaCa-2. Moreover, we observed that signal transducer and activator of transcription 3 (STAT3) phosphorylation at Tyr 705 was increased upon ligand binding in BxPC-3, but this was much less evident in PANC-1 and MIA PaCa-2 cells. Collectively, stimulation with ligand produced rapid increases of EphA2 phosphorylation, although the effects of EphA2 activation on downstream signalling differed among the pancreatic cancer cell lines. As shown in Supplementary Figure 1, neither ephrinA1 nor EphB2 were detected at significant amounts in any of the three cell lines.

### Inhibition of Src by dasatinib

**Figure 4:**
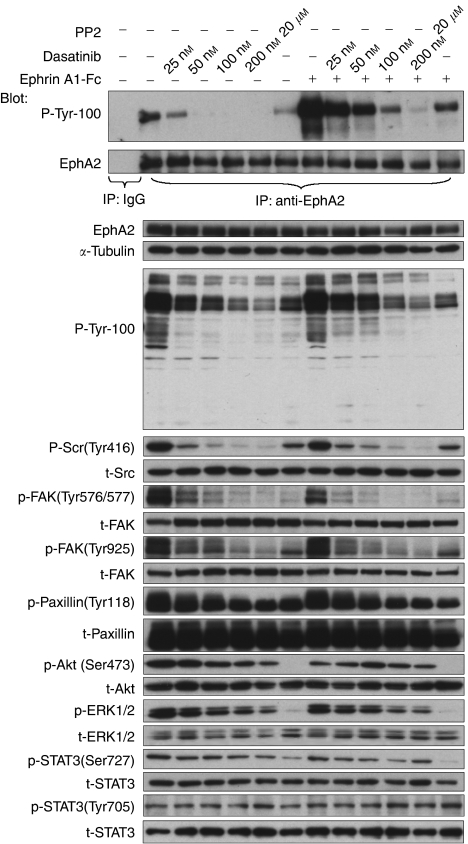
Inhibition of EphA2 receptor tyrosine kinase is dose-dependent. Around 90% confluent serum-starved BxPC-3 cells were pretreated with the indicated concentration of dasatinib or 20 *μ*M PP2 for 2 h prior to 2 *μ*g ml^−1^ ephrinA1-Fc stimulation for 5 min. Cell lysates were immunoprecipitated with anti-EphA2 antibody, analysed by phosphotyrosine (P-Tyr-100) and EphA2 immunoblots. The cell lysates were also analysed by western blot using the indicated antibodies.

Consistent with the known effect of dasatinib on Src (Serrels *et al*, 2006; Shor *et al*, 2007), Src phosphorylation at Tyr 416 and FAK phosphorylation at the Src-dependent sites (Tyr 576/577, Tyr 925) were dramatically decreased with the pretreatment of dasatinib in all three cell lines as shown in Figure 3B, and this inhibition persisted during 24 h continuous dasatinib treatment (not shown). Paxillin phosphorylation at Tyr 118 was incompletely inhibited by dasatinib. As shown in Figure 4, Src, FAK and Paxillin phosphorylations were inhibited by dasatinib in a dose-dependent manner, with or without ephrinA1-Fc ligand stimulation, and similar effects were seen with the well-characterised Src inhibitor PP2.

### Inhibition of EphA2 by dasatinib

Pretreatment with dasatinib inhibited the low levels of constitutive EphA2 tyrosine phosphorylation, as well as ligand-induced activation in all three cell lines (Figure 3A). Inhibition of EphA2 tyrosine phosphorylation was dose-dependent and the IC_50_ was similar to that for p-Src. In contrast, PP2 exhibited minimal inhibition of EphA2 tyrosine phosphorylation in BxPC-3 cells except at the highest concentration tested (20 *μ*M) (Figure 4).

### Effects of dasatinib on ephrinA1-Fc stimulation

We next examined the effects of dasatinib on the activation of downstream signalling in response to ephrinA1-Fc stimulation. In the absence of ligand, treatment with dasatinib partially inhibited Akt phosphorylation at Ser 473, ERK phosphorylation at Thr 202/Tyr 204, and STAT3 phosphorylation at Ser 727 but not Tyr 705 in all three cell lines (Figure 3B). Unexpectedly, pretreatment with dasatinib at concentrations that strongly inhibited EphA2 tyrosine phosphorylation failed to suppress completely ligand-induced activation of Akt and ERK1/2 in all three cell lines, and also the activation of STAT3 Tyr 705 that occurred in BxPC-3 cells.

### Dasatinib inhibits ligand-induced EphA2 internalisation and degradation

**Figure 5:**
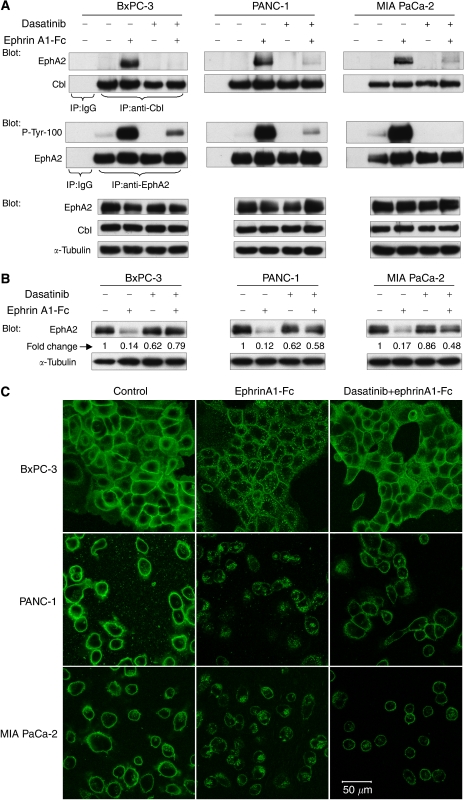
Dasatinib inhibits ligand-induced EphA2 internalisation and degradation. Approximately 90% confluent BxPC-3, PANC-1 and MIA PaCa-2 cells were pretreated with 200 nM dasatinib for 2 h prior to 2 *μ*g ml^−1^ ephrinA1-Fc stimulation. (**A**) Cell lysates were immunoprecipitated with anti-Cbl or anti-EphA2 antibody 30 min following ligand stimulation, and analysed by EphA2, phosphotyrosine (P-Tyr-100) or Cbl immunoblots. Cell lysates were also analysed by western blot using anti-EphA2 or anti-Cbl antibody. *α*-Tubulin served as the loading control. (**B**) Cell lysates probed for EphA2 3 h following ligand activation. Densitometric data (fold change) are shown, normalised to *α*-tubulin. (**C**) The subcellular distribution of EphA2 30 min following ligand activation was evaluated using confocal microscopy. Contrast enhancement was applied uniformly to all panels.

Previous work has shown that ligand-binding results in the internalisation and proteasomal degradation of EphA2, which is Cbl-dependent (Walker-Daniels *et al*, 2002; Wang *et al*, 2002). Subconfluent BxPC-3, PANC-1 and MIA PaCa-2 cells were pretreated with solvent or 200 nM dasatinib for 2 h, then stimulated with 2 *μ*g ml^−1^ ephrinA1-Fc for 30 min. In the absence of dasatinib, a large increase in EphA2 association with Cbl was observed upon ligand stimulation, concordant with the increased level of EphA2 autophosphorylation. As shown in Figure 5A, this effect was strongly inhibited by dasatinib in all three cell lines. We also observed that although there was a pronounced decrease in the total protein levels of EphA2 3 h following ligand stimulation, consistent proteasomal degradation of the receptor, this effect was diminished in the presence of dasatinib (Figure 5B). Furthermore, using confocal microscopy, we observed that the subcellular distribution of EphA2 showed internalisation followed by degradation after exposure to ephrinA1-Fc, whereas the cell surface distribution of EphA2 was maintained with dasatinib pretreatment (Figure 5C).

### Inhibition of EphA2 receptor tyrosine kinase signalling in BxPC-3 xenografts

**Figure 6:**
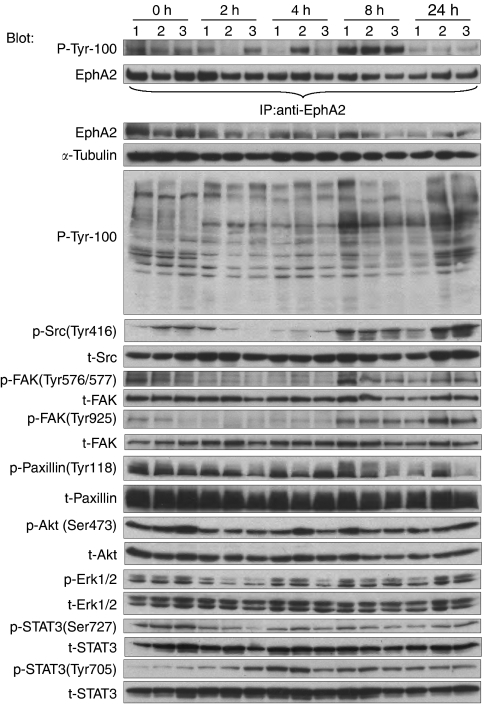
Inhibition of EphA2 receptor tyrosine kinase signalling in BxPC-3 xenografts. Mice bearing BxPC-3 xenografts were treated with single dose of 50 mg kg^−1^ dasatinib or vehicle control for the indicated periods of time. The zero time points are drug vehicle controls. Tumour lysates were immunoprecipitated with anti-EphA2 antibody, analysed by phosphotyrosine (P-Tyr-100) and EphA2 immunoblots. The tumour lysates were also analysed by western blot using the indicated antibodies.

BxPC-3 cells are the most responsive to dasatinib *in vitro* among three pancreatic cell lines, and therefore selected for the *in vivo* experiments. BxPC-3 tumour-bearing mice were treated with a single dose of 50 mg kg^−1^ dasatinib and killed at various time points. As shown in Figure 6, EphA2 tyrosine phosphorylation was readily detectable in the xenografts. This was partially inhibited after 2 and 4 h of dasatinib administration, similar to our results *in vitro*, but at the 8 h time point EphA2 phosphorylation was increased above the baseline. Phosphotyrosine proteins were decreased following dasatinib administration, but not to the extent seen in the *in vitro* studies. Src and FAK dephosphorylation occurred after 2 and 4 h of dasatinib administration as expected, and phosphorylation gradually recovered to pretreatment at 24 h, consistent with the pharmacokinetics of this compound (Lombardo *et al*, 2004). These results show that in addition to Src, EphA2 autophosphorylation is inhibited by dasatinib *in vivo*.

## Discussion

Our study focused on the inhibition of EphA2 receptor tyrosine kinase activity by dasatinib in pancreatic cancer. Although previous studies have demonstrated that dasatinib suppresses cell adhesion, migration, and invasion, and has potential as a therapeutic agent for metastatic cancers through Src inhibition (Johnson *et al*, 2005; Trevino *et al*, 2006; Shor *et al*, 2007), it seems that not all of its biological and molecular effects are due to Src inhibition (Johnson *et al*, 2005). As Src has been suggested to be involved in the activation of Eph receptors (Knöll and Drescher, 2004; Landen *et al*, 2006), we tested for direct inhibitory effect of dasatinib on Eph receptors using an *in vitro* kinase assay. The results show that dasatinib inhibits EphA2 directly, which is consistent with a recent study which reported that dasatinib has an IC_50_ of 17 nmol l^−1^ for EphA2 (Huang *et al*, 2007). Interestingly, dasatinib was also found to inhibit EphB2 directly at similar concentrations, suggesting that this agent is a more general Eph receptor inhibitor as has been suggested recently by others (Bantscheff *et al*, 2007; Rix *et al*, 2007). This is potentially important as distinct biological effects have been reported for the different Eph receptors. For example, EphB2 activity is believed to have a function in suppressing tumour progression and metastasis (Huusko *et al*, 2004; Batlle *et al*, 2005).

In this report, we detected low basal levels of EphA2 tyrosine phosphorylation in BxPC-3, PANC-1 and MIA PaCa-2 pancreatic cancer cell lines, and these were further enhanced following ligand stimulation. But the influence of Eph/ephrin activation on cell behaviour differs depending on the cell type (Kullander and Klein, 2002). Src and FAK showed transient dephosphorylation following ligand stimulation in PANC-1 and MIA PaCa-2 cells but not in BxPC-3 cells, consistent with previous studies (Miao *et al*, 2000; Duxbury *et al*, 2004), indicating that EphA2 is constitutively associated with Src and FAK, and may have an important function in regulating their functions. In contrast, paxillin, a downstream substrate of FAK, showed no obvious responses to EphA2 activation in all three cell lines.

Although Src can activate Akt directly (Jiang and Qiu, 2003) and as well affect Ras-MAPK pathway (Olayioye *et al*, 2001), activation of the EphA2 kinase has been shown to stimulate the MAPK pathway (Pratt and Kinch, 2002) and phosphoinositide 3 kinase pathway (Pandey *et al*, 1994). In contrast, others have shown that activated EphA2 downregulates the Ras-MAPK pathway (Miao *et al*, 2001). A recent study has demonstrated that EphA2 is a direct transcriptional target of the Ras-Raf-MAPK pathway, and ligand-stimulated EphA2 attenuates the growth factor-induced activation of this pathway. EphA2 signalling contributes to a feedback loop that regulates Ras activity in a ligand-dependent manner (Macrae *et al*, 2005). Our results show activation of EphA2 kinase stimulates Akt in all three cell lines. Furthermore, EphA2 activation inhibits ERK phosphorylation in BxPC-3 cells, whereas producing increased ERK phosphorylation in PANC-1 and MIA PaCa-2 cells. The explanation for this is not clear, although it should be noted that BxPC-3 cells possess a wild-type K-Ras, whereas this is mutant in PANC-1 and MIA PaCa-2.

STAT3 is a key signalling molecule for many cytokines and growth factor receptors. Transcriptional activation seems to be regulated by phosphorylation at Ser 727 through the MAPK or mammalian target of rapamycin (mTOR) pathway (Yokogami *et al*, 2000; Kanai *et al*, 2003). Although activation of STAT3 is linked to persistent activity of tyrosine kinases, including Src (Yeatman, 2004), STAT3 phosphorylation at Tyr 705, in response to cytokine stimulation, is generally mediated by Janus-activated kinase 1 (Ihle, 1995). Interestingly, we found that in BxPC-3 cells, STAT3 phosphorylation at Ser 727 was inhibited by dasatinib but unresponsive to ligand stimulation, whereas Tyr 705 was increased following ephrinA1-Fc-binding and this effect was not inhibited by dasatinib. As reported previously (Johnson *et al*, 2005, 2007), the activation of STAT3 may be a compensatory effect that suppresses the pro-apoptotic or anti-proliferative effects of dasatinib.

Dasatinib strongly inhibited EphA2 tyrosine phosphorylation of EphA2 at dose levels similar to those that inhibited Src in all three cell lines. However, as seen in Figure 2C, pronounced increases in Akt and ERK activation occurred following ligand-induced stimulation in the presence of dasatinib, whereas this was inhibited by LY294002 and U0126, respectively, as expected (data not shown). This contrasts with an extensive literature documenting that small molecule inhibitors of other RTKs such as epidermal growth factor receptor strongly inhibit ligand-induced activation of ERK and Akt. The explanation for this effect is not clear. Possibly sufficient activation of EphA2 (or perhaps a different ephrinA1-responsive receptor) persists to affect downstream signalling in the presence of dasatinib. Alternatively, higher-order clustering through the STERILE *α*-MOTIF (SAM) domain or the PDZ recognition motif might regulate ligand-stimulated signalling events (Kullander and Klein, 2002) independent of tyrosine kinase activity, and therefore insensitive to dasatinib.

Cell–cell contacts promote ligand binding, and ligand binding induces so-called ‘forward signalling’ mostly through phosphotyrosine-mediated pathways (Kullander and Klein, 2002). Upon ligand stimulation, EphA2 aggregates at the cell surface and becomes tyrosine-phosphorylated, which promotes the formation of a protein complex with the c-Cbl adapter protein (Walker-Daniels *et al*, 2002). The complex of EphA2 and Cbl is then internalised into early endosomes, where EphA2 is subsequently degraded through proteosomal and lysosomal pathways. Exposure to ligand mimetics (ephrinA1-Fc) has been shown trigger rapid EphA2 phosphorylation and receptor downregulation in MDA-MB-231 breast (Zantek *et al*, 1999; Kiewlich *et al*, 2006) and PC-3 prostate (Miao *et al*, 2000) cancer cells. We observed that dasatinib inhibited ligand-induced Cbl binding and the internalisation and degradation of EphA2, suggesting that these are dependent on kinase activity as has also been shown for kinase-dead Eph receptors (Zimmer *et al*, 2003). We suggest that through the inhibition of ligand-induced EphA2 degradation, dasatinib might act to stabilise receptor/ligand binding, thereby promoting adhesive rather than repulsive interactions, and suppressing tumour invasiveness.

Preliminary experiments showed that treatment with dasatinib results in a transient decrease of EphA2 phosphorylation in BxPC-3 xenografts, indicating that this compound can modulate EphA2 at doses that are achievable *in vivo*. Dasatinib might therefore have activity in pancreatic cancer because of EphA2 inhibition, in addition to its effects on Src.

EphA2 is overexpressed in a number of human cancers, including pancreatic cancer, and this is associated with poor prognosis. Various strategies have been proposed to target EphA2 in cancer, including the use of monoclonal antibodies directed at EphA2 surface antigens and RNAi to EphA2 (Carles-Kinch *et al*, 2002; Landen *et al*, 2005). Although it remains unclear if the activation of EphA2 tyrosine kinase is necessary for its role in cancer progression, our results suggest that more selective small molecule inhibitors of EphA2 might also have clinical value.
