# Supplementary figures and images for: Effects of dasatinib on EphA2 receptor tyrosine kinase activity and downstream signalling in pancreatic cancer
Source: Br J Cancer. 2008 Sep 16;99(7):1074–82. doi: 10.1038/sj.bjc.6604676 (PMC2567084; doi:10.1038/sj.bjc.6604676)

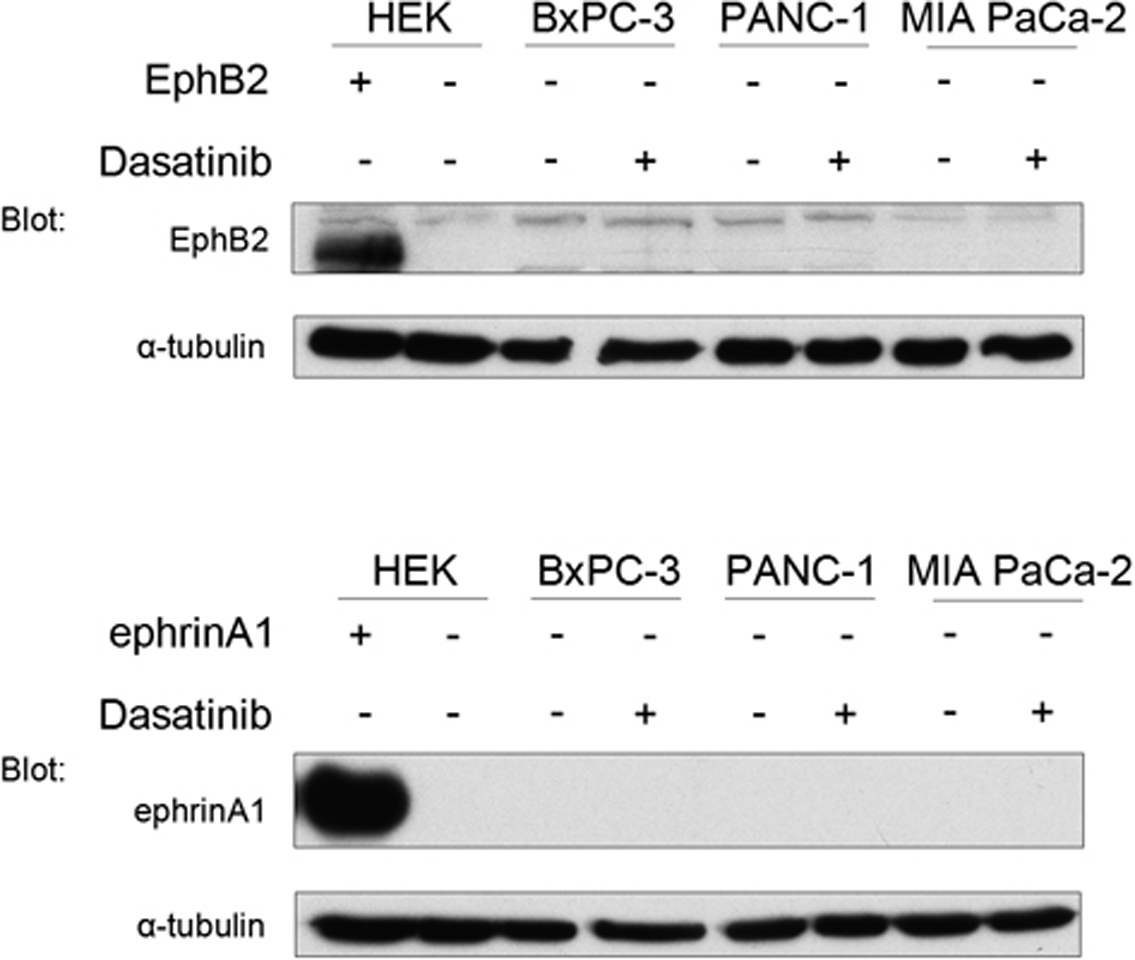

Supplement: Supplementary Figure 1 [file 6604676x1.tif]
