# Supplementary material for: Effects of dasatinib on EphA2 receptor tyrosine kinase activity and downstream signalling in pancreatic cancer
Source: Br J Cancer. 2008 Sep 16;99(7):1074–82. doi: 10.1038/sj.bjc.6604676 (PMC2567084; doi:10.1038/sj.bjc.6604676)
Supplement: Supplementary Figure 1 Legend [file 6604676x2.doc]

**Caption for supplementary figure**

**Figure Supp. 1 ephrinA1 and EphB2 expression in pancreatic cancer cell lines.**

BxPC-3, PANC-1 and MIA PaCa-2 cells were pretreated with 200 nM dasatinib for 24h.

HEK-293 cells were transfected with ephrinA1 or EphB2 constructs for 48h as positive controls. Cell lysates were analyzed by western blot using anti-EphB2 or anti-ephrinA1 antibody. α-tubulin served as the loading control.
